# Bioenergetic Profiling of Zebrafish Embryonic Development

**DOI:** 10.1371/journal.pone.0025652

**Published:** 2011-09-29

**Authors:** Krista D. Stackley, Craig C. Beeson, Jennifer J. Rahn, Sherine S. L. Chan

**Affiliations:** Department of Pharmaceutical and Biomedical Sciences, Medical University of South Carolina, Charleston, South Carolina, United States of America; Instituto de Química - Universidade de São Paulo, Brazil

## Abstract

Many debilitating conditions are linked to bioenergetic defects. Developing screens to probe the genetic and/or chemical basis for such links has proved intractable. Furthermore, there is a need for a physiologically relevant assay of bioenergetics in whole organisms, especially for early stages in life where perturbations could increase disease susceptibility with aging. Thus, we asked whether we could screen bioenergetics and mitochondrial function in the developing zebrafish embryo. We present a multiplexed method to assay bioenergetics in zebrafish embryos from the blastula period (3 hours post-fertilization, hpf) through to hatching (48 hpf). In proof of principle experiments, we measured respiration and acid extrusion of developing zebrafish embryos. We quantified respiratory coupling to various bioenergetic functions by using specific pharmacological inhibitors of bioenergetic pathways. We demonstrate that changes in the coupling to ATP turnover and proton leak are correlated with developmental stage. The multiwell format of this assay enables the user to screen for the effects of drugs and environmental agents on bioenergetics in the zebrafish embryo with high sensitivity and reproducibility.

## Introduction

Mitochondrial dysfunction is a major underlying factor in many diseases, particularly in neurological disorders that include Parkinson's disease and Alzheimer's disease. In addition, about half of the FDA approved drugs with Black Box warnings are known to cause mitochondrial dysfunction [1]. Unfortunately, the mechanisms for mitochondrial damage by these compounds are still largely unknown. Most toxicological and genetic studies that measure mitochondrial function, such as mitochondrial DNA integrity and enzymatic activities of oxidative phosphorylation proteins, are generally slow and destroy the cell or tissue of interest [2]. However, one very useful and non-invasive method to determine mitochondrial function in tissues and live cells is to measure respiration, often expressed as oxygen consumption rates (OCR, in pmol O_2_/min) [3]. Measurements of respiration are increasingly used in toxicology and several fields of biomedical research [4]–[9]. Respiration is generally measured using polarographic Clark electrodes, but these devices generally lack sensitivity and throughput. With the advent of new instrumentation, such as the Seahorse Bioscience microplate-based extracellular flux (XF) analyzer, one can continually monitor mitochondrial function in cell lines and tissues over long periods while also periodically introducing pharmacological agents to assess respiratory sources, and the coupling of respiration to ATP turnover [3],[10]–[12]. For example, inhibition of the mitochondrial ATP-synthase will assess the fraction of basal mitochondrial respiration coupled to ATP production, whereas inhibition of an ATP-consuming process will assess the fraction of respiratory ATP consumed. We reasoned that the Seahorse Bioscience XF assay could be used in combination with metabolic inhibitors to assess bioenergetics in a physiologically relevant whole organism model, the zebrafish embryo.

The zebrafish (*Danio rerio*) is an important vertebrate model organism [13], and zebrafish embryos offer many advantages over cell lines and isolated tissues for understanding basic biological processes. Breeding pairs can produce hundreds of embryos that develop outside of the mother, and thus are frequently used in high-throughput drug toxicant screens [14]–[17]. The use of zebrafish embryos and larvae for environmental agent testing is also well established [18]. Furthermore, zebrafish embryos can be genetically manipulated, and because these embryos are transparent, development can be monitored and phenotypic changes can be scored easily.

Embryonic development consists of a set of highly coordinated processes, and during these carefully timed and regulated events bioenergetic considerations are crucial. It has been shown that exposures to drugs or specific environmental conditions during the fetal period can have considerable effects on individual susceptibility to disease later in life [2], [19]–[21]. However, little is known about the long-term effects of bioenergetic perturbation during embryogenesis. A greater understanding of the metabolic partitioning of respiration would enable the examination of the possible effects drugs and/or toxicants have on the critical processes of respiration. The oxygen consumed by a tissue or organism arises from non-mitochondrial and mitochondrial respiration. Non-mitochondrial oxygen consumption can derive from many sources, such as peroxisomes and plasma membrane NADPH-oxidase activity. Mitochondrial respiration can be primarily divided into respiration due to ATP turnover and respiration due to proton leak through the mitochondrial inner membrane to the matrix [22]. The ability to measure the partitioning of total respiration among these subcategories can allow for a greater understanding of the root cause of mitochondrial dysfunction. In addition to respiration, we were also interested in determining the rate of embryonic acid extrusion (measured as proton production rates, PPR in nmol H^+^/min) into the surrounding media, and how this changes with developmental stage of the embryo. Differences in mitochondrial respiration and/or acid extrusion (i.e., metabolic acidosis) could be indicative of disruption of mitochondrial function due to xenobiotic exposures or genetic manipulations.

**Figure 1:**
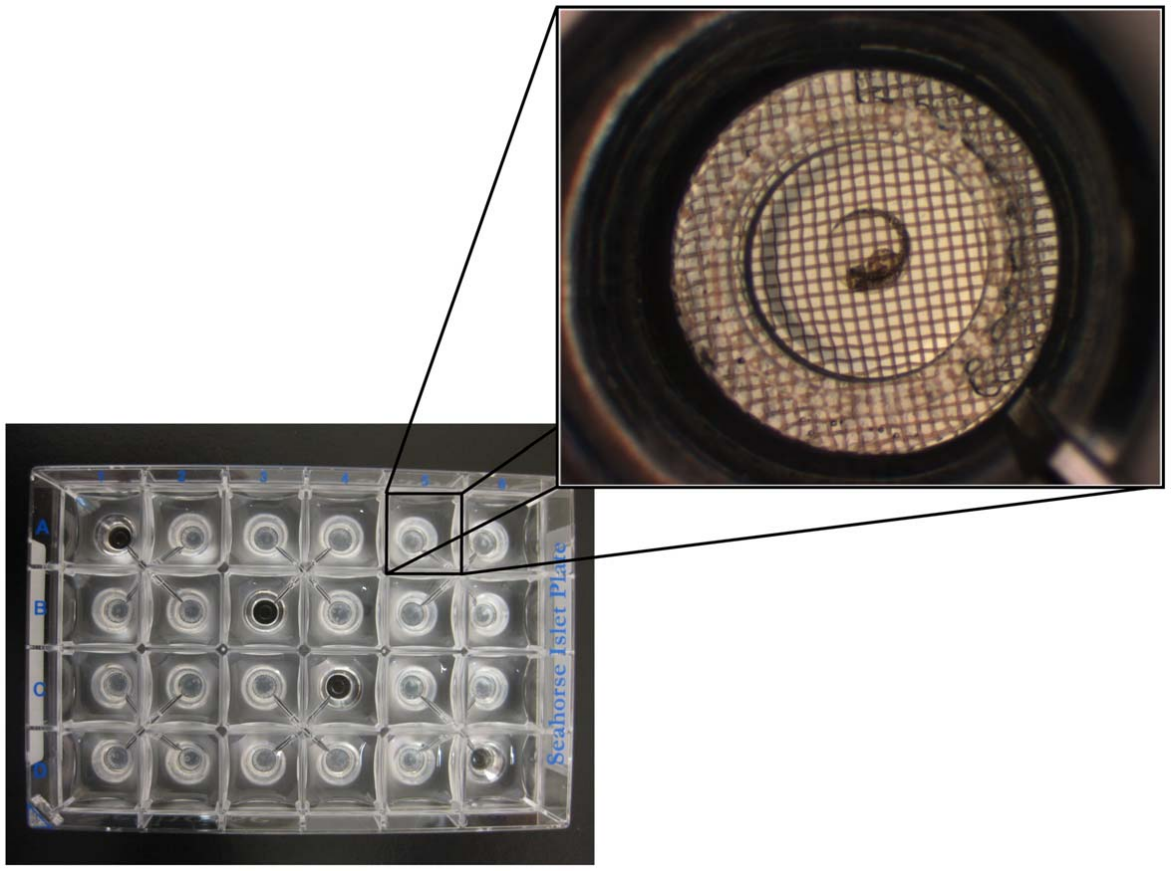
XF24 islet plate setup. Four wells were used as temperature control wells (wells A1, B3, C4 and D6) and as such contained no embryos. Embryos were plated in all other wells in numbers that allowed for respiration and media acidification measurements to be made within the recommended range. **Inset:** Expanded view of a 30 hpf zebrafish embryo in an islet plate well prior to assay.

Here we describe a novel method to screen mitochondrial function in the developing zebrafish embryo using the XF24 extracellular flux analyzer. We have adapted this platform to measure respiration and acid extrusion of developing zebrafish embryos via the use of specialized microplates where each well houses a small microchamber in which the embryos reside (Figure 1). We hypothesized that measurement of respiration and acid extrusion of zebrafish embryos at key developmental stages (e.g. blastula, gastrula, segmentation, pharyngula, and hatching periods) would enable us to monitor changes in mitochondrial function that are coordinated with embryonic development. In proof of principle experiments, we measured respiration and acid extrusion of developing zebrafish embryos. We quantified respiratory coupling to various bioenergetic functions by using specific pharmacological inhibitors of bioenergetic pathways. We demonstrate that changes in the coupling to ATP turnover and proton leak are correlated with developmental stage. Because the method employs a multiwell format, it enables screening for the effects of drugs and environmental agents on bioenergetics in the zebrafish embryo with high sensitivity and reproducibility.

## Results

### Metabolic respiration increased linearly with embryonic development

**Figure 2:**
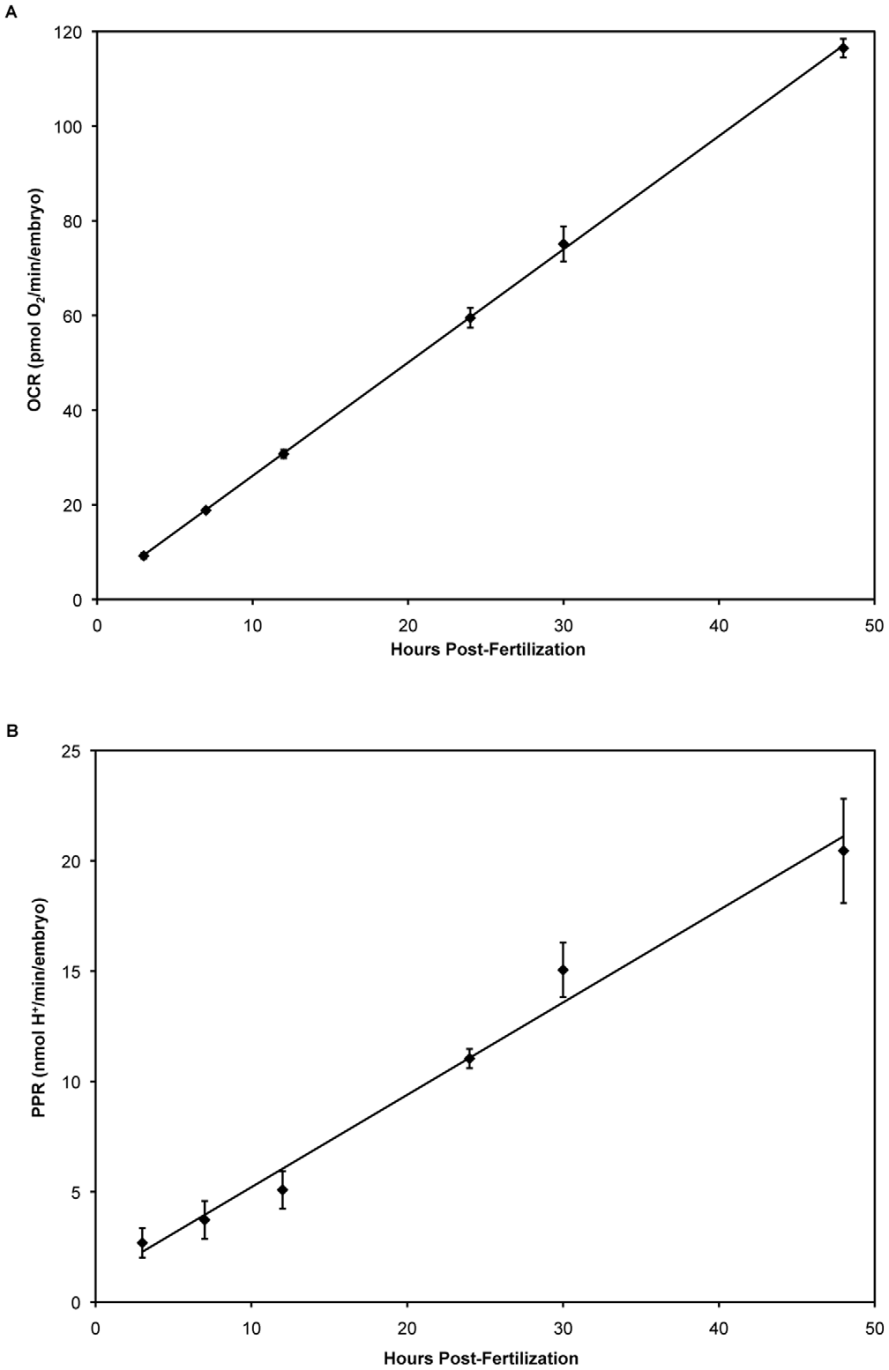
Total basal respiration and acidification of the media by zebrafish embryos. Total basal respiration (OCR, in pmol O_2_/min/embryo) and acidification of the media (PPR, in nmol H^+^/min/embryo) increased in a linear and reproducible fashion during zebrafish embryonic development (blastula (3 hpf), gastrula (7 hpf), segmentation (12 hpf), segmentation/pharyngula transition (24 hpf), mid-pharyngula (30 hpf), and hatching (48 hpf) periods). (**A**) Total basal respiration of developing zebrafish embryos (mean +/− SEM; n = 8). (**B**) Acid extrusion of developing zebrafish embryos (mean +/− SEM; n = 8).

For each XF24 multi-well islet plate, four wells were assigned as temperature control wells and did not contain any embryos (Figure 1). Embryos were placed in each of the other 20 wells, and capture screens were placed over the embryos to keep them in place (Figure 1 inset). Embryos were assayed at time-points post-fertilization corresponding to defined developmental stages (Table 1). We performed eight separate runs for each time-point, using embryos from different breeding pairs to account for possible inter-clutch differences. Basal respiration measurements were highly reproducible amongst different wells within a plate and amongst different clutches (Figure 2A). Total basal respiration increased linearly from 3 to 48 hpf (Figure 2A). Basal respiration measurements of 30 hpf embryos were also taken with a Clark electrode and the XF24 instrument in parallel to accurately compare with previous studies.

**Table 1:** Zebrafish embryos were staged at 28.5°C.

HPF	Developmental Stage	Number of Embryos
3	Blastula	3
7	Gastrula	2
12	Segmentation	2
24	Segmentation/Pharyngula Transition	2
30	Mid-Pharyngula	2
48	Hatching	1

Shown are the developmental stages and the number of embryos assayed per well.

### Embryonic acid extrusion is not primarily due to lactic acid production

Extracellular acidification rates (ECAR) expressed as mpH/min were converted to PPR expressed as pmol H^+^/min using the calculated buffer capacity and chamber volume (see Materials and Methods). As with total basal respiration, PPR increased linearly with development (Figure 2). We also observed a linear increase in PPR following treatment with FCCP (data not shown).

In cells, acidification from the media can arise predominately from glycolytic production of lactic acid, and hydration of respiratory CO_2_. In order to ascertain whether acidification of the media (egg water) was due to lactic acid production, we measured lactate levels in the media generated by embryos at all developmental stages (Table 1). Media lactate levels for all time-points were measured using a standard lactate dehydrogenase-based spectrophotometric assay [23]. Under the conditions used, lactate levels above 0.8 µM were readily detected but no lactate was detected in the zebrafish embryo media at any time-point. At 3 hpf, less than 0.1% of the PPR could be attributed to lactate. Likewise, at 48 hpf, less than 0.01% of the PPR could be attributed to lactate. Thus, the majority of acid extrusion by wild type zebrafish embryos is not due to lactic acid production.

### Metabolic partitioning of embryonic respiration

With the judicious use of pharmacological agents (Table 2), total basal respiration can be separated into its component fractions: respiration that is coupled to mitochondrial respiration (ATP turnover, proton leak) and non-mitochondrial respiration. We used oligomycin to inhibit ADP phosphorylation at the mitochondrial ATP synthase, which reveals a decrease in respiration that approximates the fraction of total basal respiration that is coupled to ATP turnover. Treatment with sodium azide blocks the respiratory chain leaving behind the fraction of total basal respiration that is non-mitochondrial. Finally, treatment with uncoupling agents such as carbonyl cyanide 4-(trifluoromethoxy) phenylhydrazone (FCCP) causes an increase in the respiration that approximates the maximal respiratory capacity when mitochondria are not substrate limited. Thus, we calculated *total mitochondrial respiration* (respiration rates after inhibition by sodium azide subtracted from total basal respiration), *respiration due to ATP turnover* (respiration rates after inhibition by oligomycin subtracted from total basal respiration), *respiration due to proton leak* into the mitochondrial matrix without associated ATP synthesis (respiration rates after inhibition by sodium azide subtracted from the respiration after inhibition of oligomycin), the *non-mitochondrial respiration* (respiration rates after inhibition by sodium azide) and *maximal FCCP-uncoupled rates* (respiration rates after inhibition by sodium azide subtracted from the respiration due to FCCP response). Each of these values are estimates, given that pharmacological agents are not 100% specific, and that any perturbation of a metabolic system will result in some type of feedback response. For example, loss of demand from the proton gradient due to oligomycin inhibition causes hyperpolarization of the inner membrane, which partly increases leak and, thus, the fraction of respiration attributed to leak is slightly over-estimated.

**Table 2:** Pharmacological inhibitors used for deconvolution of total respiration, the mechanism of action, and the final concentrations used.

Inhibitor	Mechanism	Final Concentration
Oligomycin	Inhibits the proton channel of ATP synthase, blocking phosphorylation of ADP to ATP	9.4 µM
FCCP	Mitochondrial protonophore/uncoupler, causes translocation of protons from the mitochondrial intermembrane space to the mitochondrial matrix	1.875–2.5 µM*
Sodium azide	Inhibits cytochrome c oxidase	1.25–6.25 mM#

*1.875 µM FCCP for 3–12 hpf, 2.5 µM FCCP for 24–48 hpf.

# 1.25 mM sodium azide for 48 hpf, 6.25 mM sodium azide for 3–30 hpf.

**Figure 3:**
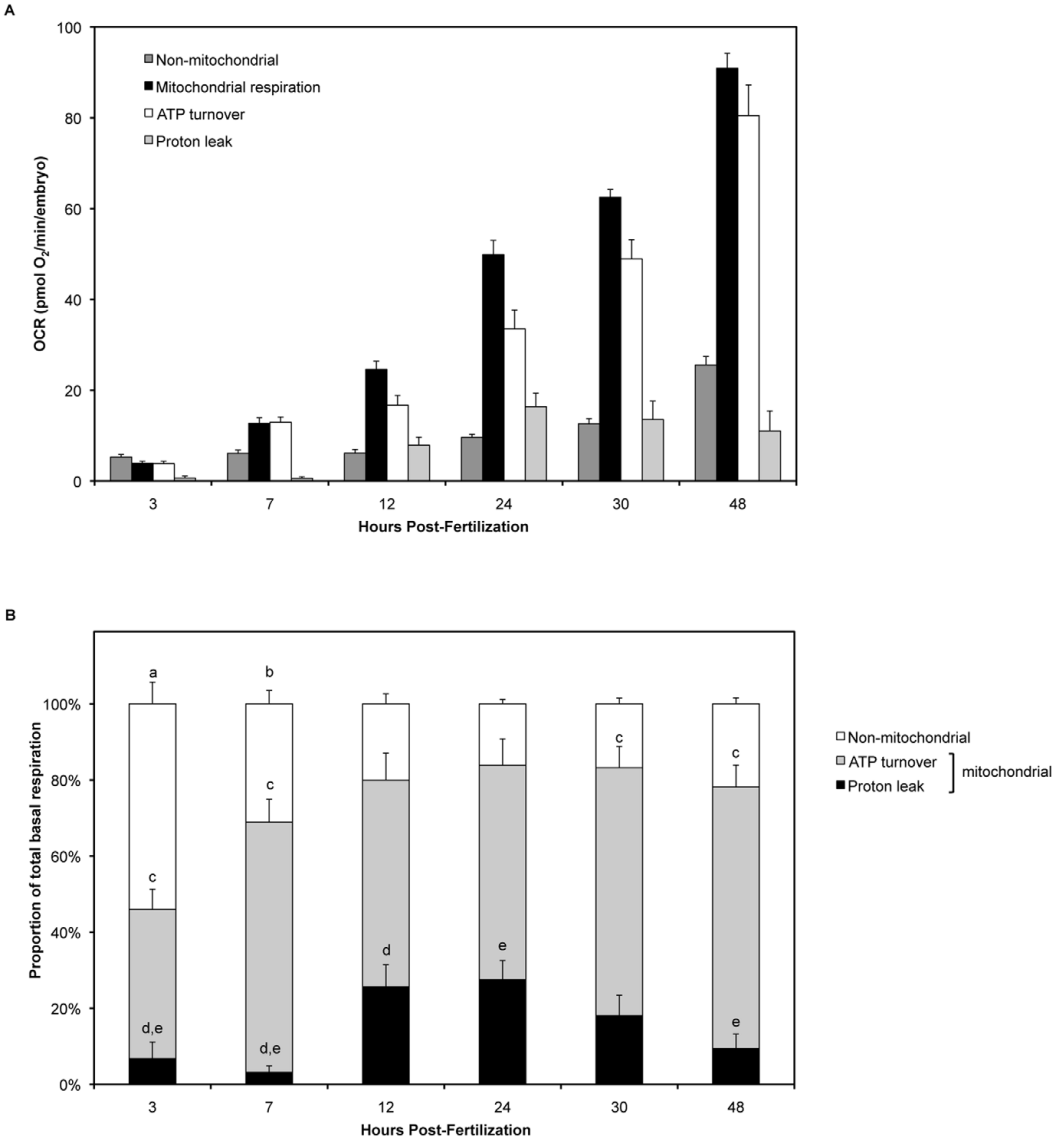
Partitioning of total basal respiration changes with zebrafish embryonic development. (**A**) The components of total respiration, per embryo. Non-mitochondrial respiration (dark grey), mitochondrial respiration (black), respiration due to ATP turnover (white) and respiration due to proton leak (light grey). Mean +/− SEM; n = 8. (**B**) Total basal respiration (100%) is composed of a non-mitochondrial fraction (white) and a mitochondrial fraction. The mitochondrial fraction is further composed of the respiration associated with proton leak (black) and ATP turnover (grey). The proportion of total respiration due to non-mitochondrial respiration was greatest at 3 hpf (p<0.0001) (a) and 7 hpf (p<0.02) (b) as determined by one-way ANOVA and Student-Newman-Keuls post hoc test. Non-mitochondrial respiration as a proportion of total respiration did not change between 12 and 48 hpf (p>0.05). At 3 hpf, the proportion of total respiration due to mitochondrial ATP turnover was lowest when compared with 7, 30 and 48 hpf (p<0.05) (c). Mitochondrial ATP turnover did not change much at other time-points. Proton leak as a proportion of total respiration at 3 hpf and 7 hpf was not significantly different to zero as determined by Student's t-test (p>0.05). Proton leak was significantly higher at 12 hpf (d) and 24 hpf (e) than at 3 and 7 hpf, as determined by one-way ANOVA and Student-Newman-Keul's post hoc test (p>0.05). Furthermore, proton leak was significantly higher at 24 hpf as compared to 48 hpf (p<0.05) (e).

Figure 3A shows how the components of total basal respiration change with developmental stage, in pmol O_2_/min/embryo. By plotting the amount each fraction of respiration contributes to total basal respiration, we can also observe how total respiration partitions with regard to developmental stage (Figure 3B). Non-mitochondrial respiration was greatest during early embryonic development (3 hpf, p<0.0001 as determined by one-way ANOVA and Student-Newman-Keuls post hoc test). Non-mitochondrial respiration gradually decreased from 3 hpf until 12 hpf, the point at which both mitochondrial and non-mitochondrial respiration remained constant throughout the rest of embryogenesis (Figure 3B). Despite the mitochondrial portion of respiration remaining constant from 12 to 48 hpf, the fractions of mitochondrial respiration coupled to proton leak and ATP turnover differed remarkably. At 3 hpf, the proportion of total respiration due to mitochondrial ATP turnover was lowest when compared with 7, 30 and 48 hpf (p<0.05). Mitochondrial ATP turnover did not change much at other time-points. Interestingly, proton leak was not statistically different from zero at 3 and 7 hpf (p>0.05 by Student's t-test). However, proton leak was greatest between 12–24 hpf (27% of total respiration) as compared with earlier stages of embryogenesis (3 and 7 hpf). The period between 10 and 24 hpf corresponds with the segmentation period in zebrafish embryogenesis. During the later developmental stages, proton leak decreased to 18% of total respiration at 30 hpf and 9.4% at 48 hpf, with proton leak at 48 hpf being significantly lower, as compared with the leak at 24 hpf.

**Figure 4:**
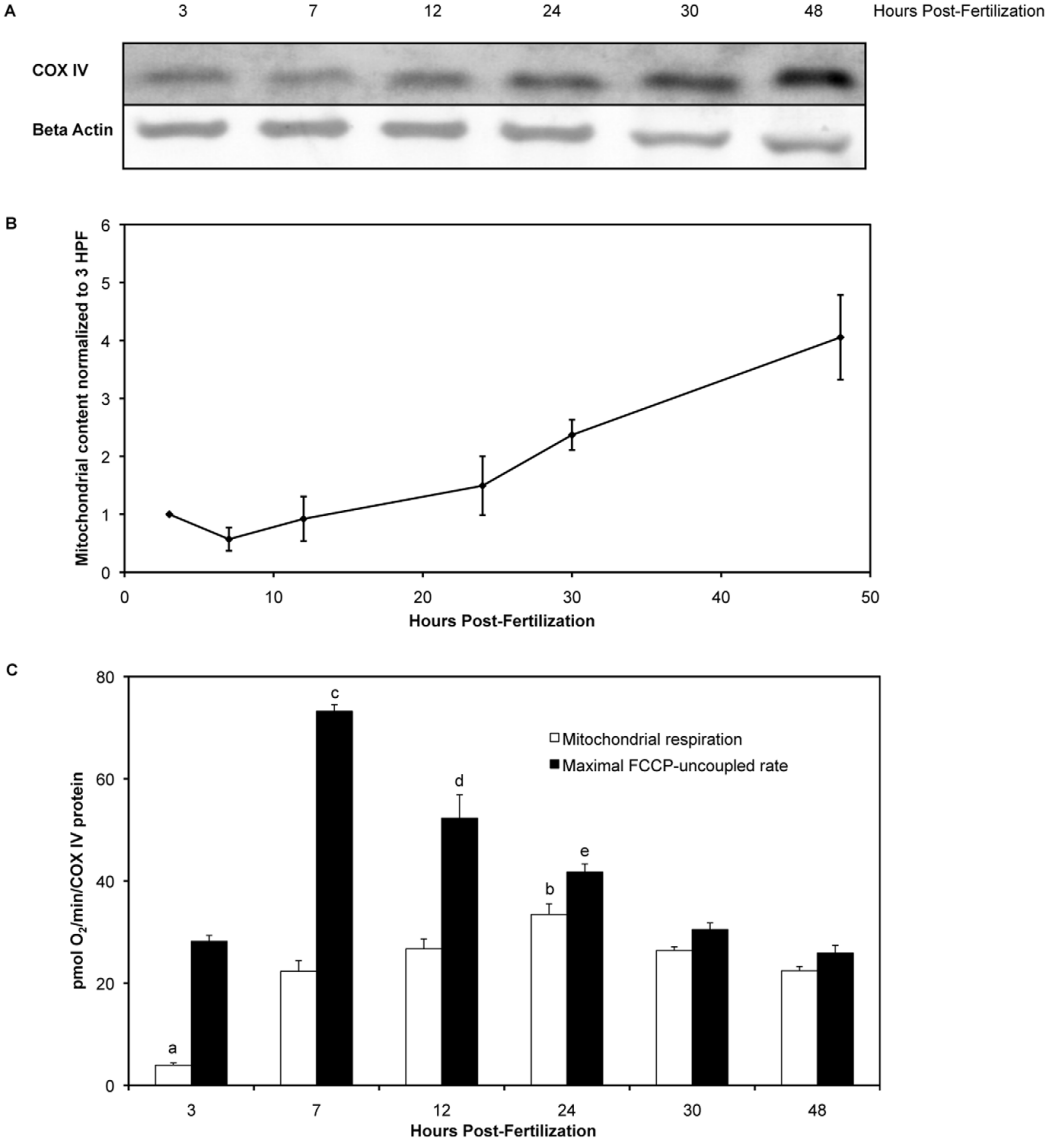
Normalization of respiration measurements by COX IV protein content (an index of mitochondrial content). (**A**) Western blots of COX IV and beta actin (loading control) proteins in zebrafish embryos aged 3 to 48 hpf. (**B**) COX IV protein levels, an index of mitochondrial content, in zebrafish embryos aged 3 to 48 hpf (mean +/− SEM; n = 3). COX IV densitometry values were normalized to beta actin values, with the value at 3 hpf set to 1. (**C**) Mitochondrial respiration (white) and maximal FCCP-uncoupled rates (black) normalized to mitochondrial COX IV protein levels (mean +/− SEM; n = 7 or 8). Mitochondrial respiration was lowest at 3 hpf, as determined by one-way ANOVA with Student-Newman-Keuls post hoc test (p<0.0001) (a). Mitochondrial respiration was maximal at 24 hpf (p<0.05) (b). However, mitochondrial respiration was similar for 7, 12, 30 and 48 hpf (p>0.05). Maximal FCCP-uncoupled rates were similar for 3, 30 and 48 hpf (p>0.05). However maximal FCCP-uncoupled rates were greatest at 7 hpf (p<0.0001) (c), followed by 12 hpf (p<0.05) (d) and 24 hpf (p<0.05) (e).

As shown in Figure 2A, total respiration increased with age of the embryo. This increase in respiration was not due to the embryo itself increasing in mass. We measured the dry mass and total protein content of thirty embryos at the assayed time-points. Neither dry mass, nor total protein changed with time. We measured levels of a mitochondrial protein, COX IV, by Western blot, with beta actin as a loading control for each time-point (Figure 4A and 4B) to obtain an estimation of the amount of mitochondria per embryo. Figures 4A and 4B show COX IV protein (an index of mitochondrial content) increasing with embryonic development. Mitochondrial respiration, when normalized to COX IV content per embryo, was lowest at 3 hpf as determined by one-way ANOVA with Student-Newman-Keuls post hoc test (p<0.0001), and maximal at 24 hpf (p<0.05) (Figure 4C). Furthermore, maximal FCCP-uncoupled rates peaked at 7 hpf (p<0.0.0001) and gradually declined with age with a plateau at 30 and 48 hpf. Mitochondrial respiration and maximal FCCP-uncoupled rates were statistically different at all time-points post-fertilization.

Because all pharmacological inhibitors have off-target effects, we verified that we have indeed measured non-mitochondrial respiration and maximal FCCP-uncoupled rates by testing additional metabolic inhibitors. For example, sodium azide is used to inhibit cytochrome c oxidase but it can also inhibit other cellular heme-based oxidases whereas other electron transport chain inhibitors such as rotenone and antimycin A can cause redox cycling. Treatment of the embryos with rotenone (a broad-spectrum piscicide and pesticide that inhibits Complex I) and antimycin A (inhibitor of Complex III of the electron transport chain) produced a similar decrease in respiration as sodium azide treatment (data not shown). Furthermore, embryos treated with dinitrophenol (a mitochondrial uncoupler) had a similar increase in respiration as observed with FCCP treatment.

## Discussion

We have fully developed a new, physiologically relevant method to assay mitochondrial and non-mitochondrial bioenergetics in the developing zebrafish embryo utilizing the XF24 instrument to make simultaneous measurements of respiration and embryonic acid extrusion. Unlike Clark electrode-based systems, the XF24 instrument sensor lowers briefly to create a transient low-volume microchamber in each well of the 24-well plate to maximize sensitivity for measurement of oxygen and proton levels. After raising the sensor and mixing, the media is re-equilibrated to enable long term, continuous measurement of respiration and media acidification, unlike the limited duration of use for a Clark electrode chamber (a closed system). The greatest advantage of the XF24 lies in the ability to easily introduce up to four pharmacological agents in a controlled environment. This is essential for our studies, where we have shown for the first time the metabolic partitioning of embryonic respiration, and how these proportions change with developmental stage in zebrafish embryos.

### Method validation

With this new method, we have shown that total respiration linearly increased with embryonic age (Figure 2). The linear increase in total respiration we observe is consistent with reports by other groups who used traditional respirometric methods to measure total respiration in fish embryos. Using Atlantic cod (a teleost model like zebrafish), Finn and Fyhn (1993) showed that the respiration of developing cod embryos and larvae increased linearly up to the hatching period [24]. Importantly, total respiration measured for zebrafish embryos at different developmental stages with the XF24 reproduce the total respiration for zebrafish embryos from 5.5–48 hpf reported by Mendelsohn *et al.*, who used a Clark-type electrode [25]. Although the Clark electrode has been the gold standard for measuring respiration [26], experiments are limited in the duration of measurement, the labor-intensive nature of processing multiple samples, as well as the difficulty of introducing pharmacological agents. These limitations are overcome with the XF24 instrument, where we were able to utilize pharmacological agents to determine the partitioning of respiration in the developing zebrafish embryo with moderate throughput.

**Figure 5:**
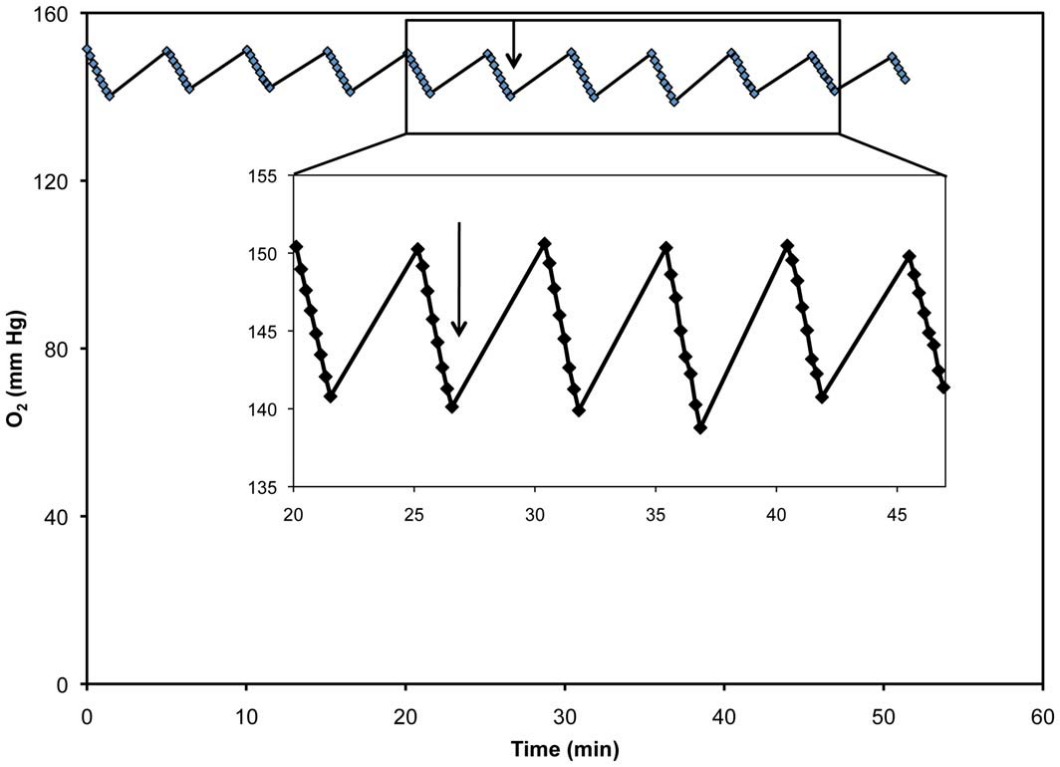
O_2_ level data for a 48 hpf embryo, as measured in the XF-24. As the sensor cartridge is lowered into the plate, a transient microchamber is formed, thus the O_2_ levels in the microchamber decrease due to oxygen use by the embryo. Arrows indicate injection of FCCP into the well. **Inset:** Expanded view of the O_2_ level data before and after FCCP injection.

In order to validate our XF24 measurements with the more commonly used Clark electrode, we performed measurements in the Clark electrode for 30 hpf embryos and used these values to compare traditional respiration data with our XF24 data. For OCR varying from 7.5–242 pmol/min, we found that the OCR per embryo measured using multiple embryos per well were equal. For example, many embryos in a single well will produce a much larger decrease in pO_2_ (mm Hg) than a well containing a single embryo, but the algorithm produces the same OCR/embryo, suggesting that its response is linear across the measured 7.5–242 pmol/min range. We also added sodium azide to these plates to show that measurements of low respiration rates were also the same per embryo regardless of the number of embryos plated. Thus, the Clark electrode calibration works similarly over the working range of oxygen concentrations. Furthermore, we looked more closely at our O_2_ level data to determine whether there were differences between slower and faster rates. We found that linear regression of these values for each rate, regardless of the type of drug injected, showed an R^2^ of 1 (Figure 5).

### Total respiration in developing zebrafish embryos

Total respiration increased with age of the embryo (Figure 2A). This increase in respiration was not due to the embryo itself increasing in mass. We measured the dry mass and total protein content of thirty embryos at the assayed time-points. Neither embryonic mass, nor total protein changed with time, and this recapitulates data reported by other researchers [24], [27], [28]. The average total OCR during the developmental stages evaluated is 3.5 nmol/h corresponding to a total consumption of 168 nmol O_2_/embryo. The loss of organic carbon mass due to substrate oxidation can thus be estimated to be no more than about 2 µg if only carbohydrates were oxidized. Given that substrate oxidation in teleost embryos involves substantial amounts of lipids [29], for which the loss of respiratory carbon is less than for carbohydrates, one would not anticipate any substantial loss of organic mass due to bioenergetic metabolism. The increase in total respiration can be attributed for the most part to the increase in mitochondrial content. We measured COX IV protein levels as an index of mitochondrial content, which increased steadily from 7 hpf onward (Figure 4B). Changes in COX IV expression without alteration in mitochondrial number are possible, however the proportions of total respiration due to mitochondrial and non-mitochondrial respiration do not change from 12 hpf onward (Figure 3B). The increase in mitochondrial respiration from 3 to 12 hpf is in keeping with the onset of mitochondrial biogenesis that occurs after the mid-blastula transition (MBT) [30].

We then analyzed the deconvoluted respiration data to understand how the various components of total respiration change with developmental stage.

### Early embryogenesis - high levels of non-mitochondrial respiration with depressed proton leak

We observed elevated non-mitochondrial respiration early in development (Figure 3). One explanation for such an observation may lie with other intracellular organelles. Enzymes within the endoplasmic reticulum, peroxisomes or plasma membrane could contribute to the high non-mitochondrial respiration [31], [32]. Bishop and Brand also suggested that the high non-mitochondrial respiration seen in hepatopancreas cells from *Helix aspersa* could be a protective mechanism to remove oxygen when it is present at potentially damaging concentrations [32]. Prior to the MBT, which starts at 3 hpf in the zebrafish embryo, cell division is rapid and there are no detectable gap checkpoints in the cell cycle [33]. Beginning at the MBT, zygotic transcription starts and the cell cycle lengthens. Thus, it is possible that this observation of elevated non-mitochondrial respiration early in embryogenesis could act as an essential mechanism for protection of DNA, protein and lipids by removing oxygen to less damaging levels. Furthermore, rapid cell division early in embryogenesis requires a large amount of energy that would best be filled by glycolysis, a process by which energy is produced at a much faster rate than through oxidative phosphorylation. Glycolysis has previously been shown to be important for very early embryonic development, with its use decreasing significantly with age [34]–[36].

At these early stages, mitochondrial respiration appears to be primarily coupled to ATP turnover with proton leak rates not significantly different from zero, as determined by Student's t-test (Figure 3B). As proton leak measurements are derived from oligomycin inhibition of respiration, and oligomycin hyperpolarizes mitochondria, our reported proton leak rates are most likely over-estimated. Thus, the low leak levels we report at 3 and 7 hpf are probably negligible, at least within the uncertainty of our measurements. Mitochondrial proton leak can be mediated by uncoupling proteins and other mitochondrial inner membrane proteins such as the adenine nucleotide translocase [37]. We speculate that since the cells of the early embryo are rapidly dividing, mitochondrial proteins such as the uncoupling proteins may not be fully functional, and this may explain the lack of measurable leak by our assay.

### Mid embryogenesis – proton leak is maximal during the segmentation period

Mitochondrial and non-mitochondrial respiration reached a plateau at 12 hpf. However, the coupling of mitochondrial respiration to proton leak and ATP turnover changed remarkably with embryonic development. We observed that the proportion of mitochondrial respiration coupled to proton leak was greatest during mid-embryogenesis, the segmentation period (10–24 hpf, Figure 3B), where 27% of the total basal respiration was attributed to proton leak. The segmentation period is characterized by the formation of somites, primary organogenesis, neuromere development, elongation of the embryo, and the first observable movements [38]. These critical processes may necessitate this observed increase in proton leak. Proton leak arises from a number of different factors that have been shown to be dependent on lipid composition of the inner mitochondrial membrane [39]. Ectotherms such as zebrafish have the capacity to modulate leak via changes in lipid composition of the inner mitochondrial membrane [40]. Mitochondria post-MBT showed negligible proton leak (Figure 3B). We hypothesize that these nascent mitochondria might not be fully developed or fully functional during the early stages post-MBT, and thus show little leak. However, later in development during the segmentation period, proton leak levels in zebrafish embryos are comparable to proton leak levels in other species, suggesting that during the critical processes of the segmentation period, mitochondria are now fully functional [41]. Additionally, proton leak could serve to regulate carbon fluxes, reduce free radical production, and increase sensitivity of key metabolic reactions to effectors [42], all of which would benefit a developing embryo during the period of segmentation.

### Late embryogenesis – oxygen depletion may cause a decrease in proton leak

Zebrafish embryos are equipped to survive only on oxygen exchange with their environment while they are enclosed in their chorion [43], [44]. Increased cardiac activity prior to hatching increases convective oxygen transport to compensate for a reduction in bulk oxygen diffusion to the tissues [28], [43], [45], and interestingly, oxygen deficits appear to initiate hatching in zebrafish [28]. At this point, proton leak may be limited to increase mitochondrial ATP synthesis efficiency.

### Maximal FCCP-uncoupled rate is greatest during gastrulation, then diminishes

Maximal FCCP-uncoupled rate, which approximates maximal respiratory capacity when mitochondria are not substrate limited, was greatest at 7 hpf, and then diminished with embryonic development (Figure 4C). As the embryo passes the MBT, zygotic transcription and mitochondrial biogenesis begin. Newly formed mitochondria would contain zygotic rather than maternal proteins. We speculate that the highest FCCP response observed at gastrulation are due to these new mitochondria. We observed that mitochondrial basal rates were close to maximal FCCP-uncoupled rates prior to hatching (Figure 4C). However, statistical analyses revealed that maximal FCCP-uncoupled rates at all time-points post-fertilization were significantly different to their corresponding mitochondrial basal respiration for each time-point. We also looked closely at our data for the possibility that oxygen limitation might be a factor in determining maximal FCCP-uncoupled rates. Well chamber conditions did not become hypoxic during the measurement period when the probes were lowered into the chamber well. Hypoxia was not found to be a contributing factor and Figure 5 shows that O_2_ levels did not drop to hypoxic conditions after injection of FCCP. Furthermore, there were no apparent limitations to either oxygen or FCCP diffusion into the embryo, as the O_2_ measurements taken to determine each rate were linear before and after injection of FCCP (Figure 5). Linear regression of these values for each rate showed an R^2^ value of 1. As zebrafish embryos are living, intact organisms able to survive in a wide range of environmental oxygen tensions, there are mechanisms in place to provide oxygen to important tissues. Unlike a similar mass of tissue taken from larger animals for respirometry, the zebrafish embryo in its later stages of development has a working circulatory system, and globin genes are expressed from 15 hpf onward in zebrafish embryos [46].

As stated previously, the increase in respiration due to treatment with the uncoupler FCCP approximates the maximal respiratory capacity when mitochondria are not substrate limited. Zebrafish embryos are supplied with nutrients from their yolk and will not exogenously feed until several days after the hatching period. Indeed, prior to hatching the embryos can deplete some of their own muscle mass to provide energy if needed [43], [47]. However, to address the question as to whether the maximum FCCP-uncoupled rates were substrate limited, we measured respiration of zebrafish embryos after addition of sodium pyruvate (1 mM and 5 mM) or octanoic acid (250 µM). Addition of these substrates did not change the total basal respiration and maximal FCCP-uncoupled rates (data not shown). External substrates such as radiolabeled pyruvate are readily taken up and metabolized by other teleost embryos such as salmon, herring and rainbow trout [47]. As the FCCP-uncoupled rates did not change despite additional substrate, this suggests that the embryos were not substrate limited. Furthermore, we observed that total basal respiration approached maximal FCCP-uncoupled rates at later stages of development, however basal respiration and maximal FCCP-uncoupled rates were significantly different at all developmental stages.

### Proton production/extracellular acidification rates in developing zebrafish embryos

This is also the first time, to our knowledge, in which acid extrusion has been measured for developing zebrafish embryos. In general, it is difficult to measure the very small pH changes surrounding small organisms or cells. However, the XF24 instrument enables highly sensitive pH measurements that are amplified by the formation of transient, low-volume chambers. The linear increase in media acidification with developmental stage is not due to lactic acid extrusion, as lactate could not be detected in the media at any stage. Despite glycolysis being a major pathway for energy production early in embryogenesis, lactate is a valuable energy resource in the developing embryo that would be best kept within the embryo itself. Previously, it had also been shown that embryos excrete undetectable levels of lactate [25], which we recapitulated by measuring lactate levels in the media at each time-point post-fertilization. As media acidification rates tracked with total basal respiration changes during development, we presume that media acidification in this case is reflective of carbonic acid production in the media due to hydration of CO_2_ generated from aerobic respiration, rather than lactic acid extrusion.

### Conclusions

We have described a new method to measure mitochondrial bioenergetics in developing zebrafish embryos. Recently, we have also initiated studies to assay respiration and media acidification of hatched larvae. These methods will allow researchers to utilize a platform to study bioenergetic metabolism in a developing organism and broaden the scope of studies that relate mitochondrial physiology and disease. This methodology could also be utilized in many diverse fields that include reproductive biology, aquaculture research, production and testing of therapeutic agents, developmental biology, and aging.

## Materials and Methods

### Ethics statement

All animal studies were approved by the Medical University of South Carolina Institutional Animal Care and Use Committee (AR #2850) and performed in accordance with the guidelines.

### Animals

Zebrafish (AB strain) were obtained from the Zebrafish International Resource Center, which is supported by grant P40 RR012546 from the NIH-NCRR. Zebrafish were maintained and crossed according to standard methods [13]. Fertilized eggs were collected and placed in egg water (60 mg/L Instant Ocean salts (Spectrum Brands, Madison, WI) in ddH_2_O), and positioned in an incubator set at 28.5°C with a 14/10 hr light/dark cycle. Embryos were staged using the criteria of Kimmel *et al.* [38]. We collected embryos at 3 hpf, 7 hpf, 12 hpf, 24 hpf, 30 hpf, and 48 hpf for experimentation. These time-points fall within the blastula (3 hpf), gastrula (7 hpf), segmentation (12 hpf), segmentation/pharyngula transition (24 hpf), mid-pharyngula (30 hpf), and hatching (48 hpf) periods in zebrafish embryonic development (Table 1).

### Pharmacological inhibitors

**Figure 6:**
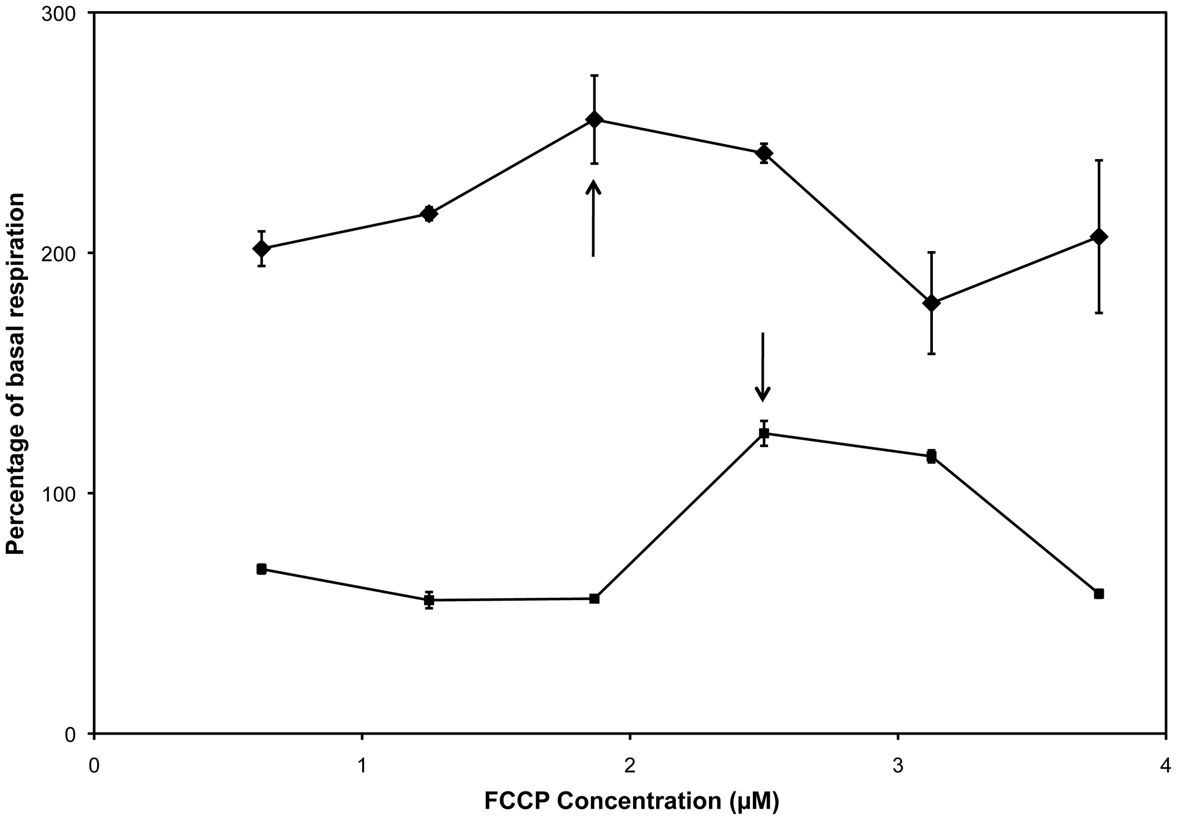
Titration curves for each agent were performed at each time-point post-fertilization. Shown here are the FCCP titration curves for 7 hpf (diamonds) and 24 hpf (squares), mean +/− SEM, n = 2-3. Arrows indicate the concentration that produced the maximum change in respiration without inducing death within the experimental timeframe.

Oligomycin, FCCP, and sodium azide were obtained from Sigma-Aldrich (St. Louis, MO). Titrations of each agent were performed at each time-point post-fertilization, to determine the concentration that produced the maximum change in respiration without inducing death within the experimental timeframe (Table 2, Figure 6). Concentrated stocks of oligomycin and FCCP were prepared in DMSO at 10 mM and 20 mM, respectively. A concentrated stock of sodium azide (5 M) was prepared in phosphate buffered saline.

### Respirometry measurements

#### XF24 analyzer

OCR measurements were performed using the XF24 Extracellular Flux Analyzer (Seahorse Bioscience, Billerica, MA). Dual-analyte sensor cartridges were soaked in XF Calibrant Solution (Seahorse Bioscience, Billerica, MA) in 24 well cell culture microplates (Seahorse Bioscience, Billerica, MA) overnight at 28.5°C to hydrate. Approximately one hour prior to experimentation, the injection ports on the sensor cartridge were filled with 100 µl of the appropriate treatments, which was then loaded into the XF24 instrument for calibration. Embryos were staged and placed into 20 of 24 wells on an islet microplate (Figure 1). Islet plate capture screens were placed over the top of the measurement area to keep the embryos in place. Four wells served as temperature control wells on each plate (A1, B3, C4 and D6). Preliminary experiments were performed to determine the number of embryos needed to achieve OCRs that fall within the recommended specifications of the XF24 instrument. The numbers of embryos used per well at each time-point are shown in Table 1. Each well was filled with 700 µL of egg water. Plates were incubated in a non-CO_2_ incubator at 28.5°C until the completion of sensor cartridge calibration. After calibration the sensor microplate was replaced with the prepared islet plate. One measurement cycle consisted of a brief wait period to acclimate the plate, 2 min mix, 1 min wait, and 1.5 min measurement. Six measurement cycles were taken to establish basal rates, which were then followed by treatment injections and 18 additional measurement cycles. OCRs were calculated using a modified AKOS algorithm that takes into account oxygen diffusion through the plate and atmospheric leak, in addition to the oxygen consumed by the organism [12]. OCR measurements were taken at cycles five and six, which were then averaged. This average value is the total basal respiration. Table 2 lists the compounds used, their mechanism, and the final concentrations used for XF24 analysis. Treatment rates were taken at two consecutive measurement cycles at the maximum OCR (after FCCP treatment) or the minimum OCR (after oligomycin or sodium azide treatment), which were then averaged. This average value is the treatment OCR. Because the AKOS algorithm as incorporated in the XF24 software is parameterized for a V7 plate, we tested whether its ability to convert O_2_ level data to OCR was linear over the range of measured rates. For OCR varying from 7.5–242 pmol/min, we found that the OCR per embryo measured using multiple embryos per well were equal. The numbers of embryos at each time-point were always adjusted to make sure that decreases in oxygen tension during rate measurements do not go below 100 mm Hg. Furthermore, as FCCP and oligomycin were prepared in DMSO, we also measured respiration of embryos treated with DMSO only. The addition of DMSO did not change respiration rates over the period of the assay.

Similarly for ECAR measurements, the fifth and sixth measurements were averaged to provide the basal ECAR, and the cycles at which the maximal or minimal OCRs were taken were used for the treatment ECAR averages.

Buffer capacity was calculated by monitoring the change in pH of the running media with 5 additions of a known quantity of protons from the addition of 0.1 N HCl. We then converted the experimentally determined ECAR to PPR by dividing ECAR by the buffer capacity. Respiration and media acidification rate measurements for embryos at each developmental stage were analyzed and normalized per embryo, as the mass of the embryo and the protein content of each embryo does not change significantly during development up to the hatching period.

#### Clark electrode

We calibrated the XF24 basal respiration using rates measured in parallel with a Clark electrode. Measurements were performed using a YSI model 5300 biological monitor and model 5331 standard oxygen probe (Fisher Scientific, Pittsburgh, PA), a 1.5 mL water-jacketed cell, a Lauda heating circulator, a magnetic stirrer, and a flat-bed recorder (Soltec, Sun Valley, CA). The instrument was calibrated according to Schnellmann [26]. Total basal respiration rates for 30 hpf embryos were measured with a Clark electrode, with an average of 0.091+/−0.004 nmol O_2_/min/embryo (n = 5 runs). In parallel, respiration was also measured in 30 hpf embryos from the same clutch using the XF24 instrument. Level data (that is, mm Hg O_2_/min/well) were converted to respiration rates (that is, pmol O_2_/min/well) using continuous averaging and the AKOS algorithm. The derived rates were then calibrated against rates measured in the Clark electrode in parallel. Using the XF24, the total basal respiration was 0.308+/0.025 nmol O_2_/min/embryo (n = 5 runs). A calibration factor was used for all XF24 OCR measurements, which was the ratio of the Clark electrode respiration rates over the XF24 respiration rates.

### Modifications for measuring respiration and acidification of the media in the XF24 instrument

Respiration and media acidification rates can also be measured for zebrafish embryos plated in V28 plates (Seahorse Biosciences) coated with Cell-Tak (BD Biosciences, San Jose, CA). We saw similar rates in the V28 plates as compared with the islet plates (data not shown). However, embryos plated in islet plates showed reduced well-to-well variances. Thus, we recommend the use of the islet plates over a Cell-Tak coated V28 plate when measuring respiration and media acidification of zebrafish embryos.

### Analysis of lactate production

Embryos were placed into individual wells of a 24 well assay plate at a density of 5 embryos per mL. Each well contained 2 mL of 0.2 µm filtered egg water. Embryos were then incubated for 1.5 hr, and 1 mL media was sampled from each well. Samples were taken in triplicate for each of the 7 time-points (3, 7, 12, 24, 30, 48 hpf). Control and treatment (sodium azide) groups were set up and run simultaneously. The sodium azide concentrations used corresponded with the concentrations used for embryos in XF24 experiments. After sodium azide was added to embryos at each time-point, embryos were incubated according to the approximate XF assay time before the media was taken for sampling. Samples of egg water were taken in 1 mL aliquots and stored at −80°C until needed. Lactate assays were performed on all samples according to the method of Brandt *et al.* [23]. All reagents were obtained from Sigma-Aldrich and solutions made fresh prior to assay.

### Western analysis of COX IV protein levels

Triplicate samples containing thirty embryos were taken at each time-point post-fertilization, and stored at −80°C until needed. For protein extraction, microfuge tubes containing thirty embryos were thawed on ice, and 150 µL cold RIPA buffer (150 mM NaCl, 1 mM EDTA, 50 mM Tris HCl pH 7.5, 1% NP-40) containing 1∶100 protease inhibitor cocktail (Sigma P8340) was added. Embryos were gently homogenized with a microfuge pestle. Tubes were rotated for 10 min at 4°C, then spun at 12,000 g at 4°C for 10 min. The supernatant was transferred to a new tube, and the protein concentration was determined by BCA protein assay (Sigma C2284 and B9643).

In each well of a 4–20% Tris-glycine polyacrylamide gel (Pierce), 30 µg of protein was loaded per time-point. After electrophoresis, proteins were transferred onto an Immobilon-P membrane (Millipore) overnight at 25 V. Western blot analysis followed established methods [48], with the following modifications. Membranes were blocked with 5% milk in TN (50 mM Tris-HCl, pH7.5, and 0.15 M NaCl), then probed using 1/1000 beta actin antibody (Sigma A2228) or 1/1000 COX IV antibody (Abcam 16056) in TN for 2 hr. Membranes were washed three times in TN, 10 min per wash. To the membrane probed with beta actin, 1/1000 goat anti-mouse HRP in 5% milk/TN was added for 2 hr. To the membrane probed with COX IV, 1/3000 goat anti-rabbit IgG HRP in TN was added for 2 hr. Membranes were washed three times in TNT (0.1% Triton X-100, 50 mM Tris-HCl, pH 7.5, and 0.5 M NaCl), 10 min per wash, then with TN three times, 10 min per wash. Novex HRP Chromogenic Substrate (Invitrogen WP20004) was added to the membrane to visualize bands. Membranes were then rinsed in ddH_2_O and dried. Blots were scanned and bands were quantified using ImageJ software (NIH). COX IV densitometry values were normalized to beta actin (loading control) values, with the value at 3 hpf set to 1. Respiration rates at each time-point were normalized to these values (Figure 4).

### Statistical Analyses

Each experiment was repeated 7–8 times with different clutches. Statistical analyses were performed using Kaleidagraph v4.0 (Synergy Software), either one-way ANOVA followed by Student-Newman-Keuls post hoc test for multiple comparisons or Student's t-test for single comparisons. Differences were considered statistically significant when p<0.05.
